# Small Bowel Epithelial Precursor Lesions: A Focus on Molecular Alterations

**DOI:** 10.3390/ijms22094388

**Published:** 2021-04-22

**Authors:** Alessandro Vanoli, Federica Grillo, Daniela Furlan, Giovanni Arpa, Oneda Grami, Camilla Guerini, Roberta Riboni, Luca Mastracci, Antonio Di Sabatino

**Affiliations:** 1 Anatomic Pathology Unit, Department of Molecular Medicine, University of Pavia and Fondazione IRCCS Policlinico San Matteo, 27100 Pavia, Lombardy, Italy; giovanni.arpa01@universitadipavia.it (G.A.); oneda.grami01@universitadipavia.it (O.G.); camilla.guerini01@universitadipavia.it (C.G.); r.riboni@smatteo.pv.it (R.R.); 2 Pathology Unit, Department of Surgical and Diagnostic Sciences, University of Genoa and Ospedale Policlinico San Martino University Hospital, 16132 Genoa, Liguria, Italy; federica.grillo@unige.it (F.G.); luca.mastracci@unige.it (L.M.); 3 Pathology Unit, Department of Medicine and Surgery, University of Insubria, 21100 Varese, Lombardy, Italy; daniela.furlan@uninsubria.it; 4 Department of Internal Medicine, University of Pavia and Fondazione IRCCS San Matteo Hospital, 27100 Pavia, Lombardy, Italy; a.disabatino@smatteo.pv.it

**Keywords:** adenoma, ampulla, *APC*, celiac disease, Crohn’s disease, *GNAS*, hamartoma, neuroendocrine, polyposis, small intestine

## Abstract

The wider use of gastrointestinal endoscopic procedures has led to an increased detection of small intestinal preneoplastic and neoplastic epithelial lesions, most of which are identified in the duodenum and ampullary region. Like their malignant counterparts, small intestinal glandular precursor lesions, which include adenomas and hamartomas, may arise sporadically or be associated with hereditary tumor syndromes, such as familial adenomatous polyposis, *MUTYH*-associated polyposis, Lynch syndrome, Peutz-Jeghers syndrome, juvenile polyposis syndrome, and Cowden syndrome. In addition, dysplastic, preinvasive lesions have been observed adjacent to small bowel adenocarcinomas complicating immune-related disorders, such as celiac or Crohn’s disease. Adenomatous lesions may exhibit an intestinal-type, gastric-type, or, very rarely, serrated differentiation, related to different molecular pathogenetic mechanisms. Finally, in the background of multiple endocrine neoplasia 1 syndrome, precursor neuroendocrine growths have been described. In this review we offer a comprehensive description on the histo-molecular features of the main histotypes of small bowel epithelial precursors lesions, including: (i) sporadic adenomas (intestinal-type and gastric-type; non-ampullary and ampullary); (ii) syndromic adenomas; (iii) small bowel dysplasia in celiac and Crohn’s disease; (iv) serrated lesions; (v) hamartomatous lesions; and (vi) neuroendocrine precursor lesions.

## 1. Introduction

The widespread use of gastrointestinal endoscopic procedures has led to increased detection of small bowel polyps, most of which are incidental lesions located in the duodenum [1,2,3]. Small bowel neoplastic lesions may be epithelial or non-epithelial. Precursor lesions, including adenomas, hamartomatous lesions, and neuroendocrine hyperplastic/dysplastic lesions, may precede epithelial malignancies, such as adenocarcinomas or neuroendocrine tumors. Small bowel (ampullary and non-ampullary) adenomas may show intestinal or gastric differentiation, as well as, very rarely, serrated morphology and may be either sporadic or related to polyposis syndromes or immune-mediated disorders [1,2,4]. The identification of such precursor lesions may have clinical implications, requiring specific endoscopic surveillance programs on the bases of their malignant potential [5] or screening of remaining organs and family members, when they are associated with hereditary tumor syndromes, such as familial adenomatous polyposis 1 (FAP), *MUTYH*-associated polyposis (MAP) or multiple endocrine neoplasia 1 syndrome (MEN1). While the molecular landscape of malignant small bowel neoplasms has been extensively studied in recent years [6,7,8,9,10,11], the genetic and epigenetic alterations of small bowel precursor lesions are less known. The aim of this review is to provide a global view of the molecular aspects of precursor epithelial lesions of the small intestine.

## 2. Sporadic Small Bowel Dysplastic Glandular Lesions

Small bowel preinvasive epithelial lesions are less common compared to their colonic counterparts. Most are located in the duodenal tract, while jejunal and ileal adenomas are rarer findings, mainly due to the fact that the distal small bowel is endoscopically less accessible, and adenomas become clinically evident only when they cause obstruction or transform into invasive adenocarcinomas. Their molecular alterations are, therefore, essentially unknown. Among duodenal lesions, ampullary lesions should be distinguished from non-ampullary duodenal adenomas (NADAs).

### 2.1. Non-Ampullary Duodenal Adenomas

**Figure 1:**
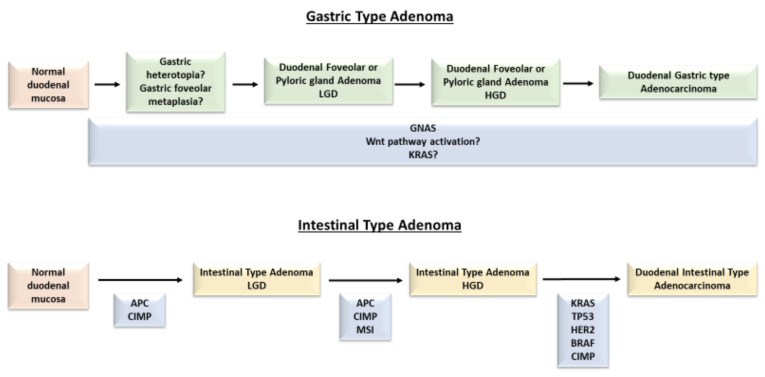
Mutational path from dysplastic precursor lesions to duodenal adenocarcinoma (gastric type and intestinal type). *APC*: adenomatous polyposis coli; *BRAF*: v-raf murine sarcoma viral oncogene homolog B1; CIMP: CpG island methylator phenotype; *GNAS*: guanine nucleotide binding protein, alpha stimulating; *HER2*: human epidermal growth factor receptor 2; HGD: high grade dysplasia; *KRAS*: kirsten rat sarcoma; LGD: low grade dysplasia; MSI: microsatellite instability; *TP53*: tumor protein p53; Wnt: wingless-related integration site.

According to the literature data, sporadic NADAs seem to account for approximately 40% of all NADAs. Most sporadic NADAs are asymptomatic and identified in elderly men (60–80 years old) [2,12]. Histologically, NADAs are classified as intestinal-type and gastric-type according to their histologic phenotype [4]. Interestingly, the two histologic subtypes seem to arise through separate carcinogenetic molecular pathways (Figure 1).

#### 2.1.1. Intestinal-Type Adenomas

**Figure 2:**
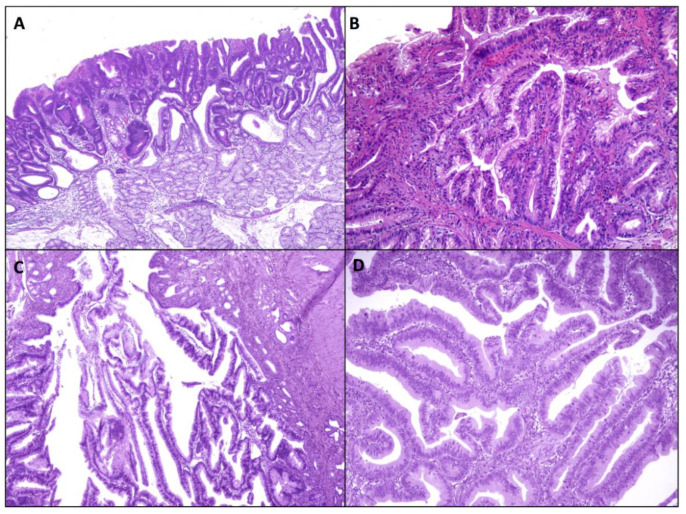
Non-ampullary and ampullary sporadic, glandular precursor lesions. (**A**) Sporadic non-ampullary, low-grade, intestinal-type adenoma. (**B**) Sporadic non-ampullary, low-grade foveolar-type adenoma. (**C**,**D**) Sporadic non-invasive intra-ampullary papillary-tubular neoplasm, with admixed low grade (**C**) and high grade (**D**) dysplastic foci. Patients signed informed consent regarding publishing their data before having their endoscopic/surgical procedure.

Intestinal-type lesions are the predominant subtype among sporadic NADAs [2,13] and their preferential location is the second portion of the duodenal tract. They are morphologically defined as the dysplastic transformation of the small bowel epithelial cells, similar to that seen in colorectal conventional adenomas; a luminal brush border and goblet and/or Paneth cells are generally found. Histologically, low grade adenomas are composed of crowded and closely packed cells with elongated, hyperchromatic nuclei which involve the entire thickness of the mucosa replacing normal cells (Figure 2A). They may show either a tubular or tubular-villous architecture. High grade dysplastic lesions, defined as distorted, back-to-back or cribriform glands, marked nuclear stratification and more severe atypia with enlarged hyperchromatic nuclei with prominent nucleoli and loss of nuclear polarity, are identified in up to 40% of cases. Nevertheless, some studies reported that intestinal-type NADAs have a lower potential for malignant transformation compared to gastric-type NADAs [12,14]. Immunophenotypically, intestinal-type NADAs are characterized by CD10, CDX2, and/or MUC2 protein positivity. However, a focal gastric differentiation, supported by MUC5AC and MUC6 expression, may be present in otherwise typical intestinal-type adenomas [12,13,14].

While little is known on the molecular features of jejunal or ileal adenomas, the most frequent genetic alterations of intestinal-type NADAs involve *APC* and *KRAS* genes, similar to colorectal adenomas [15,16]. In a recent study, Ota et al. performed integrated genetic and epigenetic analysis of 107 NADAs and intramucosal adenocarcinomas, comprising 100 intestinal-type neoplasms (90 adenomas, 10 carcinomas) [16]. Their molecular analysis showed that *APC* was the most commonly mutated gene (55%), followed by *KRAS* (13%).

Overall, Wnt/β-catenin signaling pathway has been reported to be dysregulated in NADAs [15,16,17,18]. Ota et al. found *APC* mutation in 53%, 59%, and 60% of low grade intestinal-type NADAs, high grade intestinal-type NADAs and intestinal-type intramucosal adenocarcinoma, respectively [16]. Most *APC* mutations are nonsense or frameshift mutations, mainly distributed in the mutation cluster regions (between codons 700 and 1200 or between codons 1400 and 1600), though a few missense mutations have also been identified. The two more common mutational hotspots within *APC* gene in NADAs are T1556fs and R1450X [16,19]. Alterations in mutational cluster regions seem to be more common in intestinal-type NADAs (86%) in comparison with intestinal-type intramucosal adenocarcinomas (33%) [16]. In addition, *APC* alterations have been found to be rarer in advanced duodenal carcinomas [8,9,10,11,19,20], suggesting that most NADAs have a low potential for malignant progression to duodenal adenocarcinomas and that the adenoma-carcinoma sequence may play a small role in the development of invasive duodenal adenocarcinomas.

Several studies found a high prevalence (64–90%) of extensive or focal nuclear β-catenin expression in intestinal-type NADAs, as well as alterations in Wnt pathway components, indicating the relevance of Wnt signaling for the development of NADAs [15,16,17,18]. Among intestinal-type NADAs, the prevalence of β-catenin accumulation does not seem to significantly differ between low grade and high-grade lesions, even if Niwa et al. [17] observed that nuclear β-catenin was more frequent in high-grade lesions and larger size (>20 mm in diameter) NADAs (93%), which are associated with a higher risk of progression to adenocarcinoma [5]. Interestingly, the frequency of nuclear β-catenin accumulation has been reported to be significantly higher in intestinal-type neoplasms compared to gastric-type tumors [16], suggesting a more limited contribution of Wnt pathway in the pathogenesis of duodenal adenomas and adenocarcinomas with gastric phenotype.

*KRAS* mutations were found in 18% of NADAs by Wagner et al., while they seem to be more common in ampullary adenomas (44%), likely as a result of the presence of bile and pancreatic secretions in the periampullary region [15]. Interestingly, in the study by Matsubara et al., *KRAS* mutation was less frequent among intestinal-type adenomas in comparison with gastric-type adenomas with pyloric gland phenotype [21]. As *KRAS* mutations are significantly rarer in low grade intestinal-type NADAs (3%) and high grade intestinal-type NADAs (7%) in comparison with intestinal-type intramucosal adenocarcinomas (40%), they seem to be related to neoplastic progression [16].

*GNAS* mutations (mainly at codon 201) have never, or very rarely, been identified in intestinal-type NADAs [16,21]. In contrast, both *GNAS* and *KRAS* alterations were significantly more common in gastric-type neoplasms [16].

*BRAF* alterations have been described in 6% of low grade intestinal-type NADAs and in 3% of high-grade intestinal type NADAs [16]. *BRAF* V600E mutation has never been described in NADAs, while it may be present in up to 10% of *BRAF*-mutated small bowel adenocarcinomas [8].

*ERBB2* mutations occur in less than 5% of NADAs, while the combined prevalence of mutations or copy number gains in any members of ERBB receptor family may reach 34% [16]. *ERBB2* mutations and/or amplifications have been detected in up to 23% of small bowel adenocarcinomas and they are associated with duodenal location and microsatellite instability [8,9,10,11,20,22]. Interestingly *ERBB2* inhibitors have been found to display anti-cancer activity in small bowel adenocarcinomas, both in vitro and in vivo [10].

Microsatellite instability has been reported to be absent or very rare in NADAs [15]. Matsubara et al. [21] found only one intestinal-type adenoma that exhibited a loss of mismatch repair (MMR) protein expression (loss of MLH1 and PMS2), while Ota et al. observed no sample with loss of MLH1 immunoreactivity [16]. In duodenal adenocarcinomas microsatellite instability may be associated with *MLH1* methylation [23], however, epigenetic alterations of NADAs are poorly characterized. *MLH1* methylation has been recently observed in only 2% of intestinal-type NADAs/intramucosal adenocarcinomas, without a significant association with CpG island methylator phenotype (CIMP) [16]. In particular, *MLH1* methylation was found in 3% of high grade and in no low grade NADAs by Ota et al. and in 12% of NADAs as a whole by Sun et al. [24]. CIMP was observed in 16% of low grade NADAs and in 24% of high grade NADAs, while intramucosal adenocarcinomas showed higher rates of CIMP, suggesting a role of such an epigenetic alteration in NADA progression.

*TP53* abnormalities are infrequent among intestinal-type NADAs [15]. In particular, *TP53* mutations have been identified in only 5% of NADAs by Ota et al. [16], while recent studies indicate that *TP53* (38–58%), *KRAS* (27–54%), and *APC* (11–27%) are the most frequently mutated genes in small bowel adenocarcinomas [9,10,11,20], suggesting that this molecular alteration is a late event in tumorigenesis.

#### 2.1.2. Gastric-Type Adenomas

Gastric-type adenomas represent about 10% of all small bowel adenomas and have been described almost exclusively in the duodenum [2]. They include pyloric gland adenomas and foveolar adenomas [12,14]. Duodenal gastric-type adenomas are mainly found in the proximal duodenum and tend to show higher rates of malignant transformation in comparison with intestinal-type adenomas.

Duodenal pyloric gland adenomas histologically and molecularly resemble their more frequent gastric counterpart [25,26]. They consist of closely packed glands lined by a single layer of cuboidal or low columnar cells, with pale-to-eosinophilic, “ground-glass” cytoplasm and round nucleus, resembling pyloric glands. They are typically immunoreactive for MUC6, the pyloric/Brunner gland mucin marker, while foveolar marker MUC5AC expression is usually limited to the surface epithelium. However, mixed-diffuse staining for both MUC6 and MUC5AC throughout the lesion may be observed.

In a recent analysis of 57 cases of duodenal pyloric gland adenomas, they were more frequently detected in the duodenal bulb/proximal tract of elderly individuals and showed polypoid, nodular or plaque-like endoscopic patterns, with an average size of 15 mm [26]. By definition, all pyloric gland adenomas exhibit at least low-grade architectural and cytological dysplastic features. High-grade dysplasia, with cribriform or back-to-back glandular structures, lined by highly atypical cells, has been found in around 40% of cases, while association with invasive cancer has been reported in a variable fraction of cases ranging from 17% to 66% [26,27]. Tumor size (lesions larger than 2 cm) and architectural pattern (villous) have been found to be related with high-grade dysplasia or adenocarcinoma [26].

Molecular alterations of pyloric gland adenomas have been poorly investigated. Hida et al. found mutation in guanine nucleotide-binding protein alpha subunit (*GNAS*) gene in 4 out of 7 (57%) duodenal adenomas with gastric phenotype, and 2 of the 5 cases (40%) of pyloric gland adenomas, whereas *APC*, *BRAF*, *KRAS,* and *CTNNB1* genes were wild-type in all investigated gastric-type adenomas [27]. These findings support the hypothesis that *GNAS* mutation contributes to the pathogenesis of pyloric gland adenomas of the duodenum, as well as those of the stomach. Mutations in other oncogenes, like *KRAS* or in oncosuppressor genes like *SMAD4* and *TP53*, have been observed by other authors [16,28].

*GNAS* gene located at chromosome 20q13.32 is responsible for encoding the G-alpha subunit (Gsα) of the heterotrimeric guanine nucleotide-binding proteins (G-proteins), which transduce signals from G protein-coupled receptors (GPCR) to adenyl cyclase by releasing guanosine diphosphate (GDP) and combining with guanosine triphosphate (GTP), and finally resulting in protein kinase A activation by cyclic adenosine monophosphate (cAMP) and in transcription of several genes involved in tumorigenesis and regulation of mucin expression and secretion [29,30]. In the case of missense mutations of *GNAS* gene, such as those resulting in R201H and R201C variants, the encoded protein is constitutionally active. Among gastrointestinal tract neoplasms, such *GNAS* gene alterations have been found in pancreatic intraductal papillary mucinous neoplasms (mostly of intestinal type) and colloid carcinomas [31,32,33], low-grade appendiceal mucinous neoplasms/pseudomyxoma peritonei [30], intestinal villous adenomas [34], in addition to gastric and duodenal pyloric gland adenomas and duodenal adenocarcinoma [16,21,27,35]. Interestingly, in duodenal adenocarcinoma, *GNAS* mutation was associated with gastric phenotype [35], suggesting that pyloric gland adenomas might represent the precursor lesions of duodenal adenocarcinomas with gastric differentiation. Worthy of note, *GNAS* molecular alterations have not been reported in intestinal-type adenomas of the duodenum [21].

Despite the rarity of *APC* or *CTNNB1* mutations, duodenal pyloric gland adenomas have been reported to express nuclear β-catenin (a marker of Wnt pathway activation) by immunohistochemistry in a variable fraction of cases (up to 80%), suggesting that currently unknown molecular alterations other than *CTNNB1* or *APC* mutation may be responsible for Wnt pathway activation. The protein kinase A, activated by *GNAS* mutations, might play a role in Wnt pathway activation in pyloric gland adenomas by stabilizing β-catenin [36].

Although the histogenesis of duodenal pyloric gland adenomas is poorly known, there is some evidence that at least a fraction of them (up to 26%) may arise in a background of gastric heterotopia, which might represent its precursor lesion [26,27,37,38]. Indeed, molecular changes reported in pyloric gland adenomas, such as *GNAS* and *KRAS* gene alterations, were also found in areas of gastric heterotopia of the duodenum. In particular, Matsubara et al. described *GNAS* and *KRAS* gene mutations in 28% and 2% of gastric heterotopias, and in 17% and 37% of duodenal adenocarcinomas, respectively, supporting a pathogenetic link between gastric heterotopias and gastric-type adenomas or duodenal adenocarcinomas [35]. In addition, Matsubara et al. found *GNAS* and *KRAS* mutations in 41% and 26% of gastric foveolar metaplastic lesions of the duodenum, in keeping with other authors. This finding suggests that a fraction of gastric-type adenomas and duodenal adenocarcinomas might arise from gastric foveolar metaplastic epithelium in damaged duodenal mucosa with Brunner gland hyperplasia, which may accumulate mutations over time and develop dysplastic or cancerous changes [37,39,40,41]. In contrast, genetic mutations were reported to be rare among gastric foveolar metaplastic lesions associated with active inflammation, supporting a reactive nature of these lesions [35], and the rarity of duodenal non-ampullary adenocarcinomas, in contrast to the relatively frequent presence of gastric foveolar metaplasia in the proximal duodenum, suggests that cancer development from foveolar metaplastic lesion, though possible, is a rare event.

One case of duodenal pyloric gland adenoma associated with suspected Lynch syndrome (LS) has been reported, while Mc-Cune-Albright syndrome, characterized by germ-line mutations of *GNAS*, does not seem to predispose to the development of such duodenal lesions [26]. In contrast to their gastric counterpart, no case of duodenal pyloric gland adenoma has yet been described in the setting of hereditary polyposis [26]. Further investigations are, however, needed to estimate the actual prevalence of gastric-type duodenal adenomas in hereditary gastrointestinal tumor predisposing syndromes. Interestingly, one case occurred in a patient with celiac disease (CD), an autoimmune condition with a higher risk of developing small intestine adenomas and adenocarcinomas [42].

Duodenal foveolar adenomas are extremely rare polypoid lesions, featuring a tubulo-villous architecture (Figure 2B). Such foveolar adenomas are composed of tall columnar dysplastic cells, with a Periodic-Acid-Schiff (PAS)-positive and MUC5AC-positive apical cytoplasmic mucin cap, thus morphologically and immunophenotypically resembling gastric foveolar cells. MUC6-reactive cells may be present; however, they are usually scattered, at variance with pyloric gland adenomas. They should be distinguished from atypical foveolar metaplasia. Unfortunately, molecular features of these adenomas are currently poorly known, due to their rare occurrence. In one study including two foveolar adenomas of the duodenum, both harbored *GNAS* mutations, while no *APC*, *KRAS*, or *CTNNB1* mutations were found [27]. These findings suggest that the two subtypes of duodenal adenomas with gastric phenotype may be molecularly close to each other; further studies are needed to support this idea.

### 2.2. Ampullary Preinvasive Neoplasms

The ampulla of Vater is a complex region which opens into the duodenal lumen and is characterized by the convergence of diverse anatomic structures. It includes 3 main types of epithelium: distal bile duct epithelium, pancreatic ductal epithelium, and small bowel epithelium on the duodenal surface of the papilla. Ampullary adenocarcinomas can therefore be classified into 3 distinct histological subtypes according to morphologic and immunophenotypical characteristics: intestinal, pancreato-biliary, and mixed type, with possible prognostic and molecular differences, although this has not been reproduced by all research groups [43,44,45,46]. While the molecular landscape of ampullary adenocarcinomas has been extensively studied [47,48], and has shown alterations in five major signaling pathways (*TP53*/cell division, *RAS/PI3K*, Wnt, TGF-β, and chromatin remodeling pathway) with similarities and differences between intestinal and pancreato-biliary phenotype [49,50], much less is known about the molecular alterations in its precursor lesions. Two main pre-invasive lesions have been identified: intestinal-type adenomas developing from the overlying ampullary duodenal mucosa and intra-ampullary papillary neoplasms (IAPN) developing from, and expanding, the ampullary channel.

#### 2.2.1. Ampullary Duodenal Adenomas

Ampullary duodenal adenomas (ADA) involve the duodenal surface and may develop sporadically or in hereditary syndromes, such as FAP [51]. The ampulla shows a higher frequency of such lesions, compared to non-ampullary duodenal mucosa, possibly due to the prolonged exposure of the ampullary mucosa to bile, pancreatic secretions, and the intestinal microbiota. ADAs are histologically very similar to their colorectal counterparts and they may show low- and high-grade dysplasia. They are considered non-invasive precursor lesions in the adenoma-carcinoma sequence of some ampullary adenocarcinomas.

ADAs, both sporadic and FAP-associated, show dysregulation of the oncogenic Wnt signaling pathway. Mutations in the *APC* gene have been found in adenomas and in early stage ampullary adenocarcinomas, identifying this alteration as an early event. Furthermore, sporadic ADAs have been shown to differ from those of FAP, with regards to *APC* somatic mutation prevalence (17% vs. 64%) and site of mutation, suggesting a distinct molecular pathogenesis for the two conditions [52]. Loss of heterozygosity (LOH) at 5q21, where the *APC* gene is located, has been demonstrated in 70% of sporadic ampullary tumors [53], comprehensive of 75% of adenomas and early-stage cancers, and this also suggests its contribution in the early phase of carcinomas development.

With regards to the MAP kinase pathway, *KRAS* mutations have been described in about 40% of ampullary adenocarcinomas [54] as well as in ADAs (about 30%), even in areas of low-grade dysplasia [55,56]. Importantly, *KRAS* mutations have been found in a high percentage of ampullary adenomas (93%) when they are adjacent to invasive carcinomas [56,57,58]. This finding supports the concept that adenomas are precancerous conditions and that *KRAS* alterations occur at an early stage of ampullary cancerogenesis. *BRAF* is much less frequently mutated in ampullary tumors, and when found, it is more likely to affect intestinal type-ampullary adenomas [55]. No mutation has been identified in the *HRAS*, *NRAS*, and *PIK3CA* loci.

The TGF-β pathway has also been demonstrated to be involved in ampullary cancerogenesis [59], however, while loss of expression of *SMAD4*, an oncosuppressor gene located at 18q21.1 and involved in growth inhibition, has been identified in 34% of invasive ampullary cancers, none of the associated adenomas (except for focally in high grade dysplasia) showed such loss [60]. This suggests that *SMAD4* loss probably represents a late genetic alteration in ampullary tumorigenesis.

Microsatellite instability (MSI) has been demonstrated in ampullary carcinoma in a variable number of cases in the literature, ranging from 6% to as high as 22% [61,62]. The largest study on ampullary adenomas and adenocarcinomas showed MSI-H in 9% of adenomas (9/89) and in 10% of adenocarcinomas (15/144) with high levels of MSI concordance between the adenomatous component and adjacent invasive component, when present [63]. A third of MSI-H adenomas showed *MLH1* promoter hypermethylation while a further third of such adenomas showed loss of *MSH6* (and could be part of LS). MSI is probably, therefore, an early alteration which develops at the stage of intestinal-type ADA.

#### 2.2.2. Intra-Ampullary Papillary-Tubular Neoplasms

Described in 2010, IntraAmpullary Papillary-Tubular Neoplasms (IAPN) are preinvasive exophytic tumors which grow almost exclusively (>75%) within the ampulla (within the ampullary channel or in intra-ampullary segments of the distal common bile or pancreatic duct) (Figure 2C,D). These neoplasms are predominantly preinvasive but are often (78%) associated with an invasive adenocarcinoma component [64] which drives prognosis (5-year survival of non-invasive vs invasive lesions: 100% vs. 45%). Importantly, invasive carcinomas associated with IAPN have a better survival compared to cancers associated with flat dysplasia (5-year disease free survival IAPN associated vs flat dysplasia associated cancers: 70% vs. 50%) [65]. No specific information on the molecular background of this subtype of pre-invasive lesion is yet available.

## 3. Small Bowel Adenomas in Hereditary Syndromes

Inherited predisposition to small bowel adenomas is mainly associated with adenomatous polyposis syndromes and LS. To date, two major inherited monogenic forms of adenomatous polyposis are recognized: an autosomal dominant form FAP [MIM: 175100], due to heterozygous germline mutations in the oncosuppressor gene *APC* [66], and an autosomal recessive form MAP [MIM: 608456], due to biallelic constitutional mutations in the base-excision-repair gene *MUTYH* [67,68]. Recently three new entities of adenomatous polyposis syndromes have been described—the autosomal dominant polymerase proofreading-associated polyposis syndrome, caused by mutations in *POLD1* (MIM:174761) and *POLE* (MIM: 174762) genes [69,70], the autosomal recessive *NTHL1*-associated polyposis (MIM: 616415) [71], and the autosomal recessive *MSH3*-associated polyposis (MIM: 600887).

Adenomatous lesions of the ampullary or non-ampullary duodenum can be detected in 30–70% of FAP individuals, who show a lifetime risk of 88% for duodenal polyposis and 18% for duodenal adenocarcinoma [72,73,74,75].

In FAP patients, adenomas can be seen throughout the whole duodenal tract, even though the second and third portions and the ampulla are the most involved sites. Duodenal/periampullary adenocarcinoma is the most frequent extracolonic malignancy in FAP and the third main cause of death among these patients (8.2%). Indeed, these individuals harbor a 100–330-fold higher risk of duodenal adenocarcinoma in comparison with the general population [76]. The average age at adenocarcinoma diagnosis has been reported to be 47–51 years old [77,78].

The first endoscopic examination should be carried out between 25 and 30 years of age or just before colectomy. The Spigelman classification is associated with the risk of duodenal adenocarcinoma development, and its score, based on polyp number, size, histologic features and grade of dysplasia, is useful to guide endoscopic follow-up [79,80]. Patients with stage IV polyposis according to the Spiegelman classification exhibit a 36% risk of duodenal cancer within 10 years and surgical management including pancreaticoduodenectomy is suggested for such patients [81].

Moreover, FAP individuals are at risk for ileal adenomas, especially after total proctocolectomy with ileal pouch-anal anastomosis, with a risk as high as 75% 15 years after surgery [66] and are counselled to undergo periodic endoscopic surveillance [79,82].

Attenuated FAP represents a phenotypically different FAP variant, also due to an *APC* mutation. Attenuated FAP patients characteristically harbor less than 100 colorectal adenomas, lower overall risk, and later development of cancer [66]. This syndrome is also associated with duodenal adenomas and similar cumulative risk of small intestinal adenocarcinoma development as FAP patients. For these reasons, upper gastrointestinal surveillance should likewise be performed in attenuated FAP patients.

Despite the established role of endoscopy and surgery in FAP management, nonsteroidal anti-inflammatory agents have been used to control colorectal adenoma development and delay the need for surgery [83,84,85]. Such a chemoprevention strategy has also been applied to duodenal polyposis, characterized by limitations in endoscopic resection techniques and relevant surgery-associated morbidity [86]. This approach has not, as yet, shown a significant role in the management of duodenal polyposis [87,88]. Notwithstanding this, a recent promising clinical trial using a combination of cyclooxygenase and epidermal growth factor receptor inhibition found a significant regression (70%) of duodenal adenomas after 6 months of therapy [89].

Both FAP and attenuated FAP are caused by germline or somatic mosaic mutations in *APC*, a tumor suppressor gene which plays a pivotal role in the Wnt signaling pathway, especially in the degradation of β-catenin. When *APC* is inactivated, the β-catenin-Tcf complex is not degraded, and this leads to constitutive activation of molecular pathways involved in cell growth and proliferation [75]. *APC* gene, which is located on chromosome 5q22 and includes 15 coding exons, translates to a protein comprising 2843 amino acids [75]. About 30% of all *APC* germ line alterations occur at codons 1061 and 1309, while the remaining mutations are spread rather uniformly between codons 200 and 1600.

**Figure 3:**
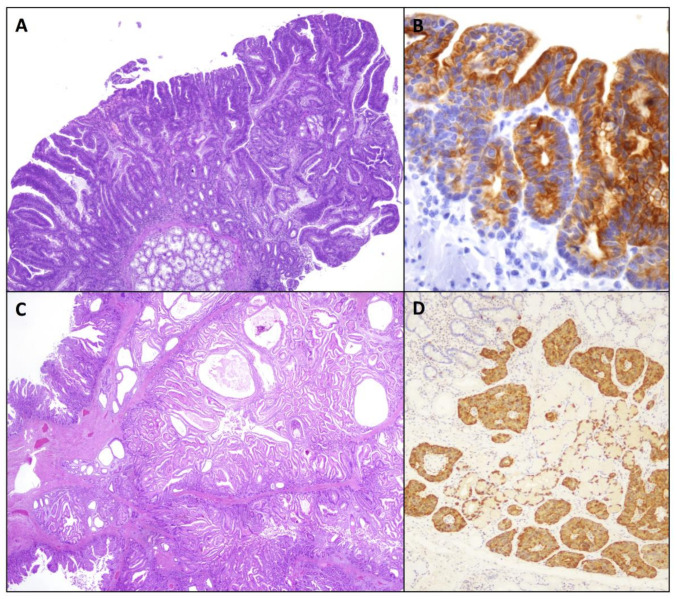
Precursor epithelial (glandular and neuroendocrine) lesions occurring in a background of hereditary tumor syndromes or immune-mediated disorders. (**A**) *MUTYH*-associated polyposis syndrome-associated non-ampullary duodenal adenoma showing high grade dysplasia. (**B**) Cytokeratin 7 positive ileal flat dysplasia adjacent to a small bowel adenocarcinoma associated to Crohn’s disease. (**C**) Peutz-Jeghers polyp. (**D**) Gastrin cell preneoplastic lesions in a patient affected by multiple endocrine neoplasia type 1 syndrome-related duodenal gastrinomas, including enlarged nodules and microinvasive lesions, in close relationship with linear and nodular gastrin cell hyperplasia of Brunner’s glands. Patients signed informed consent regarding publishing their data before having their endoscopic/surgical procedure.

MAP is also associated with duodenal adenomas (Figure 3A), found in 17–34% of patients undergoing endoscopy, and the lifetime risk of duodenal adenocarcinoma has been estimated to be about 4% [90,91]. Recent guidelines recommend similar endoscopic follow-up programs for both FAP and MAP [82,92,93]. However, it has been suggested that present recommendations using Spigelman staging may not be appropriate for MAP patients. Indeed, duodenal adenocarcinomas in MAP can be observed in the absence of advanced polyposis of the duodenum, since Spigelman stage IV polyposis occurs rarely in MAP cases [94,95]. Thomas et al. [96] recently demonstrated a higher mutational burden in MAP compared to FAP duodenal adenomas. These observations suggest that the risk of progression of duodenal adenomas to adenocarcinoma is likely higher in MAP compared to FAP, challenging the assumption that the same surveillance strategies should be applied to both syndromes. The greater burden of somatic alterations and copy number variations observed in MAP adenomatous growths is the result of base excision repair defects in MAP patients [96,97]. The *MUTYH* gene, which is located on chromosome 1 (1p32.1–p34.3) and includes 16 exons, encodes for a glycosylase involved in base excision repair system and in repairing DNA mutations secondary to oxidative DNA damage. More than 300 *MUTYH* pathogenic variants have been described in MAP individuals. The mutation spectrum varies according to ethnic groups, suggesting population specific ancestral variants [98]. The Collaborative Group on MAP reported that the risk of duodenal polyposis in these patients is related to genotype, showing that p.Y179C homozygote patients have an increased risk [99,100].

*APC* and *MUTYH* gene analysis are required by diagnostic protocols in the background of inherited adenomatous polyposis; furthermore, mutations in *POLE*, *POLD1*, *NTLH1*, and *MSH3* genes are nowadays also screened by most laboratories.

Polymerase proofreading-associated polyposis, named PPAP is a recently described condition, related to the susceptibility genes *POLE* and *POLD1*, which encode for DNA polymerases with proofreading activity. To date, *POLE* and *POLD1* germline mutations have been correlated with attenuated colorectal polyposis, while gastroduodenal adenomatous growths have been observed in about 57% of carriers [70].

The most recently identified polyposis syndromes are *NTHL1*-associated polyposis and *MSH3*-associated polyposis [71,101].

*NTHL1* is a base excision repair gene and its involvement in inherited adenomatous polyposis was discovered through exome sequencing on 51 individuals with multiple colorectal adenomatous polyps, negative for *APC* and *MUTYH* germline mutations. Germline homozygous or compound heterozygous *NTHL1* mutations have been associated with multiple colorectal adenomas but also with adenomas in extracolonic sites, including the duodenum.

Similarly, Adam et al. [101] carried out whole exome sequencing on patients with unexplained adenomatous polyposis, and found two unrelated individuals showing different compound heterozygous mutations in *MSH3*, a gene involved in the MMR system. Interestingly, *MSH3* mutation carriers may also feature duodenal adenomas.

LS is an autosomal dominant syndrome due to a germline mutation of MMR genes and is related to an increased risk of large bowel carcinoma and of other neoplasms including small bowel cancer [102]. The MMR genes, which are involved in post-replicative proofreading, include *MLH1* (on chromosome 3p21), *MSH2* and *MSH6* (on chromosome 2p16), and *PMS2* (on chromosome 7p22). In addition, deletion in the *EPCAM* gene may lead to *MSH2* inactivation. As a consequence of impaired MMR activity, MSI phenotype, characterized by alterations in the length of tandem repeats within microsatellites, develops.

Recent investigations have analyzed the life-time risk of small intestinal neoplasms in LS patients, and they found an average risk of 4–5% [103]. Numerous case series have described small bowel cancers in LS patients and demonstrated that these neoplasms can be the first and only cancer in this syndrome. Moreover, these patients may develop malignancy as soon as the early teens and most tumors are located in the duodenum or the jejunum [104,105]. The pathologist may have an important role in identifying LS patients through the routine use of MMR immunohistochemical analysis and MSI testing in both precursor lesions and cancers of the small bowel. Small bowel surveillance in LS remains a controversial subject. Video capsule endoscopy has been suggested as a possible tool to screen LS individuals for tumors of the small intestine [106].

## 4. Premalignant Epithelial Lesions in Celiac Disease and Crohn’s Disease

As yet, scarce knowledge regarding precancerous lesions in chronic immune-mediated intestinal conditions, such as celiac disease (CD) and Crohn’s disease (CrD), is available, mostly because of their rarity. Nevertheless, both CD and CrD are recognized risk factors for the development of small intestine adenocarcinoma [107].

### 4.1. Celiac Disease

In CD, both raised/adenomatous preinvasive growths and flat dysplastic lesions have been described, always adjacent to carcinoma [108,109,110]. It was recently demonstrated that small bowel adenomas are detected much more frequently in CD patients than in non-CD individuals, with a relative risk of 5.73 [42]. However, in the mucosa adjacent to small bowel carcinoma, flat dysplasia, albeit rare, seems to be more frequent than adenomatous growths [110]. In the small intestine of CD patients, the relevance of the adenoma-carcinoma sequence is therefore still unclear. Translocation of β-catenin, and of Wnt-pathway related Sex-determining Region Y-Box (SOX) 9 in nuclei, have been determined to be characteristic features of both precancerous lesions and early changes in the carcinogenic process of CD-associated small bowel carcinoma, while p53 overexpression has been described in 80% of preinvasive lesions adjacent to cancer [110]. Moreover, both in cancer-adjacent and cancer-distant small intestine mucosa of CD patients, foci of relatively immature SOX-9-positive crypt hyperplasia, often with topographic continuity with the invasive neoplasm and associated with the other typical CD inflammatory changes, have been demonstrated, suggesting, in such a condition, the possibility of an inflammation-hyperplasia-dysplasia-carcinoma sequence [107,110].

On the contrary, MSI due to *MLH1* hypermethylation, which is a common feature of CD-associated small bowel adenocarcinoma [9,110,111,112] appears to be a late event in small bowel carcinogenesis in these patients, as it was solely detected in the preinvasive component of one case [110].

### 4.2. Crohn’s Disease

CrD-related small bowel dysplastic lesions have been found in about half of cases of CrD-associated small bowel adenocarcinoma, more often adjacent to the invasive component [113] (Figure 3B). In addition, rare dysplastic lesions without cancer may be found in the ileum of CrD patients and they are usually flat and low-grade. CrD-related small bowel dysplastic lesions can be raised, flat, or mixed (raised and flat) and arise almost exclusively in the setting of an inflamed mucosa [113]. Histologically, CrD-associated dysplasia is usually conventional/adenomatous, resembling conventional colorectal adenomas; however, recently, in the colorectum of inflammatory bowel disease patients, various non-conventional histological variants of precursor, preinvasive growths have been identified [114,115,116]. Some non-conventional lesions have been rarely seen adjacent to CrD-associated small bowel adenocarcinomas, such as the “hypermucinous” type, characterized by a villiform architecture composed of columnar cells with mucin-rich cytoplasm and small or slightly enlarged basally oriented nuclei, and the “eosinophilic” type, characterized by a tubular or tubulo-villous architecture mainly composed of enterocyte-type cells with enlarged, hyperchromatic, mildly to severely atypical nuclei and only a few goblet-cells [114].

CrD-related dysplasia characteristically shows p53 overexpression at a high rate (47–59%), in keeping with the frequent detection of *TP53* mutations in CrD-associated small bowel adenocarcinomas [8,11,113,117]. These findings indicate a key role of *TP53* gene in the initial steps of small bowel carcinogenesis of CrD patients. On the other hand, an infrequent nuclear localization of β-catenin in both CrD-associated dysplasia and adenocarcinomas compared to CD patients has been reported, suggesting a more limited role of canonical Wnt pathway deregulation in CrD-associated small bowel carcinogenesis. Nevertheless, non-canonical Wnt pathways, which are activated by CrD pro-inflammatory cytokines, could be involved in CrD-associated small bowel carcinogenesis [107,110].

Similarly to CD-related dysplasia, microsatellite instability seems to play a minor role in early phases of the carcinogenic process in CrD-associated lesions also [107,110,113]. Molecular studies have described *KRAS* mutations in a small number of CrD-associated preinvasive growths (about 13%), found in exon 12 or, in only a single dysplastic lesion, in exon 37 [113,114]. *PIK3CA* mutations were also found, mostly in “eosinophilic” type dysplasia [114]. On the contrary, no *BRAF*, *NRAS*, or *EGFR* mutations were reported [113].

Interestingly, the vast majority (>90%) of CrD-associated dysplastic lesions exhibit a metaplastic phenotype, highlighted by MUC5AC and/or by cytokeratin 7 expression; the same phenotype can be also identified in non-dysplastic, inflamed mucosa of patients harboring a CrD-small bowel carcinoma. These findings hint at the possibility of a distinct histogenetic process in CrD patients, operating through the inflammation-metaplasia-dysplasia-carcinoma sequence [107,110].

## 5. Serrated Lesions

Three subtypes of serrated polyps are recognized in the colorectum, namely hyperplastic polyps (Hyp-P), sessile serrated lesions, and traditional serrated adenomas (TSA). While serrated polyps in the colorectum are quite frequent, with up to 30% of adenocarcinomas developing through the serrated pathway, they are rarely described in the small bowel, and almost all are localized in duodenum.

Few reports on Hyp-Ps are available in the international literature, as case reports or small case series [118,119,120]. Confusion concerning these entities is subsequent to problems with nomenclature, since the major part of duodenal Hyp-Ps (2.8%—16/615—in consecutive duodenal specimens [121]) resemble hyperplastic polyps of the stomach, arising in the context of gastric metaplasia, and not microvesicular intestinal hyperplastic polyps of the colon. Furthermore, in the manuscript by Liu [122], 37 cases of inflammatory/hyperplastic polyps of the small bowel are described, which are mostly sporadic (28 cases) while 9 are found in the context of various syndromic conditions, in particular juvenile polyposis syndrome (JuvPS). Only 6 out of 37 cases showed a pure intestinal type, while gastric epithelial differentiation was present in all except one sporadic Hyp-P, making it difficult to translate these findings to intestinal Hyp-Ps.

Hyp-Ps of the small bowel, morphologically resembling colorectal Hyp-Ps, are typically small, sessile, asymptomatic and incidentally discovered lesions, which usually involve the second part of the duodenum [2]. Histologically, they are characterized by hyperplastic, columnar mucinous epithelium showing serration and lacking cytologic atypia. Molecular testing information, available for 8 Hyp-P from 2 different studies [119,120], has demonstrated *KRAS* mutation in 2, *BRAF* V600E mutation in 2, and no mutation for either gene in the remaining 4 polyps. Even though few cases have been analyzed, the molecular profile of duodenal Hyp-Ps seems to overlap with that of colorectal Hyp-p, which also harbor *BRAF* V600E or *KRAS* mutations.

A little more information is available on TSA of the small bowel. Since the first report by Rubio in 2004 [123], several studies have been published [124,125,126,127,128,129], reaching a comprehensive number of about 40 cases. Small intestine TSAs are typically localized in the second portion of the duodenum or in the papilla. Their microscopic features overlap with TSAs of the colorectum, being characterized by eosinophilic tall cells with penicillate nuclei, prominent serration, and ectopic crypt foci [125]. High grade dysplasia (with both serrated and conventional features) has been recorded in about half of the cases [124]. Although slow growing, their behavior is aggressive with almost 28.6% progressing to adenocarcinoma [126]. Data on the molecular profile of these lesions derives from the largest published series of 13 cases, where 50% of tested cases (6/12) showed CpG island methylation phenotype (CIMP-high) and 38% (6/13) *KRAS* mutation, while no *BRAF* V600E mutation or lack of MLH1 expression were described [124]. Interestingly, unlike colorectal TSAs where the CIMP phenotype is concomitant with *BRAF* mutation, in duodenal TSAs, CIMP phenotype is an early and frequent molecular event unrelated to MLH1 loss and *BRAF* mutations. This perhaps represents an alternative pathway of serrated carcinogenesis, which will require further demonstration and analysis on a greater number of small bowel TSAs.

## 6. Hamartomatous Lesions

Hamartomas are defined as non-neoplastic tumor-like proliferations of normal tissue with mature cells, in excess and disordered with respect to their normal counterparts. Hamartomatous polyps of the small bowel can be solitary (or few) and sporadic or, more frequently, multiple as part of hereditary hamartomatous polyposis syndromes: Peutz-Jeghers Syndrome (PJS), JuvPS, and PTEN Hamartoma Tumour Syndrome. The latter includes several conditions characterized by mutations in *PTEN*, a gene implicated in tumor suppression, the most common of which is Cowden Syndrome (CS). Polyp site and macroscopic/microscopic aspects vary between different conditions, but the overlap of histologic aspects can cause difficulties in the prompt recognition of different hamartomatous polyposis syndromes.

Polyps in PJS are more frequently diagnosed in the small bowel (60–90%), where they are responsible for the majority of gastro-intestinal PJS symptoms, followed by the large bowel (25–50%) and, more rarely, the stomach [130]. On the other hand, less than 10% of sporadic, JuvPS and CS polyps affect the small bowel, while the largest quota (about 75%) is localized in the colon and rectum [131].

Peutz-Jeghers polyps show a pedunculated and lobulated shape, with variable size but often in the form of large and exophytic masses. Microscopically, they are characterized by an arborizing proliferation of smooth muscle fibers within the lamina propria and glands are often dilated and filled with mucus (this last aspect is more pronounced in Juvenile polyps) (Figure 3C). Erosion is usually absent or scarce (but this greatly depends on polyp size) while there is a stromal mixed inflammatory infiltrate. Their clinical presentation includes bleeding and bowel obstruction due to intussusception [132].

Juvenile polyps may vary greatly in size, but they are usually large and exophytic with marked surface erosion. They always show glandular distortion with dilated cystic glands filled with dense mucus, markedly inflamed stroma with granulation tissue and scarce smooth muscle proliferation and arborization. Their morphological aspect often overlaps with inflammatory polyps and distinction can prove difficult, if not impossible. Common reported symptoms comprise rectal bleeding, anemia, and abdominal discomfort. Of note, albeit called “juvenile”, these polyps can be diagnosed at any age.

CS polyps are characteristically small, multiple and sessile, without erosion, and with mild inflammation admixed with fibrosis, moderate smooth muscle proliferation and lymphoid follicles. Gland distortion and dilatation is present only in a minority of cases and dense intraluminal mucin is absent [131]. It is important to underline that in CS, multiple polyp types can be recognized other than hamartomatous polyps, including traditional adenoma, hyperplastic polyps, ganglioneuroma [133], and fibroblastic polyps [134].

Sporadic polyps share most of their features with syndromic juvenile polyps. They are usually large, exophytic, eroded masses with inflammatory infiltrate and cystic dilatation of glands containing thick mucus; sporadic polyps are more frequently encountered in the large bowel, exceptionally in the small bowel and almost never in the stomach [131].

Patients with hamartomatous polyposis syndromes show a high risk of gastrointestinal and/or extra-gastrointestinal cancer, starting from a young age, while no increase of cancer risk has been demonstrated in patients with sporadic hamartomatous polyps nor in their relatives [135,136].

The progression to cancer through hamartomatous polyps is still controversial. The ‘landscape’ hypothesis suggested in 1998 by Kinzler and Vogelstein [137] suggests that the microenvironment created by abnormal stromal proliferation promotes malignant transformation of the adjacent epithelial cells via cell regeneration due to damage, thus leading to the hamartoma-adenoma-carcinoma sequence proposed by Bosman [138]. This hypothesis was based on the demonstration of genetic alterations in stromal cells of syndromic juvenile polyps but not in the epithelial component. Several authors have tried to consolidate this hypothesis by studying genetic alterations in the stroma and epithelium of both syndromic juvenile and Peutz-Jeghers polyps, with inconclusive and contrasting results [139,140,141,142].

*Peutz-Jeghers Syndrome*—PJS is an inherited autosomal dominant syndrome occurring at a variable rate, affecting between 1/50,000 and 1/200,000 individuals, characterized by mucocutaneous maculae along with hamartomatous polyps and increased risk for gastrointestinal, breast, testicular and gynecological cancers varying between 10 and 50% (13% for small bowel cancer). About a quarter of patients with PJS are “de novo” cases. More than 90% of patients fulfilling the diagnostic criteria for PJS and carry mutation in *STK11*, a tumor suppression gene [143], located at 19p13.3, which encodes for a serine/threonine kinase. *STK11* contributes to controlling different processes such as cellular metabolism and proliferation, cellular polarity and apoptosis, controlling AMP-activated protein kinase family members, and downregulating the mammalian target of rapamycin (mTOR) pathway [144]. Missense/nonsense mutations, mutations in splicing sites, small insertions, deletions, and indel type mutations cover the majority (about 70%) of *STK11* alterations in PJS [145]. The remaining 30% of cases are represented by rearrangements for deletions, insertions, or combined mutations of larger fragments of the *STK11* sequence. Patients with clinical diagnosis of PJS and with wild-type *STK11*, have shown heterozygous mutation in a great variety of other genes, including *APC* and DNA MMR genes [146]. Mutation type does not seem to correlate with cancer risk [147] while, more recently, it has been suggested that hypomethylation of *STK11* promoter in PJS polyps might represent a risk factor for gastrointestinal malignancy development [148].

*Juvenile Polyposis Syndrome*—JuvPS is an autosomal dominant disease affecting 1:100,000 new births per year [149] and is clinically suspected in the presence of 5 or more colorectal juvenile polyps or any non-colorectal juvenile polyp or any polyp in association with a family history of JuvPS. On the basis of polyp site and age at presentation, four different JuvPS subtypes have been recognized: (i) JuvPS of infancy, manifesting within two years of life with frequently fatal prognosis; (ii) a form in which polyps are limited to colon and rectum; (iii) a form in which polyps are limited to the stomach; (iv) and a form in which polyps are distributed throughout the gastrointestinal tract. JuvPS patients have a high risk (86%) of malignancy, more frequently gastric and colorectal carcinoma. Risk of developing a small intestine cancer is very low (1.6–2.3%), limited to JuvPS subtypes with only colorectal polyps or with a disseminated polyposis in the gastrointestinal tract [150]. Germline mutations in *SMAD4* (18q21) and *BMPR1A* (10q22-23) gene, both part of the transforming growth factor-beta (TGF-β) signaling pathway, have been detected in about 20–30% of cases respectively [151], as point mutations and, less frequently, large deletions [152]. The TGF-β pathway is involved in cellular growth, differentiation, homeostasis, and apoptosis. Other than *SMAD4* and *BMPR1A*, the *ENG* gene, encoding a membrane glycoprotein participating in TGF-β pathway, has also been suggested to rarely contribute to JuvPS [153], although its role is still uncertain [154]. Patients carrying *SMAD4* mutations have an increased risk of developing juvenile polyps in the stomach and manifest symptoms later than patients with *BMPR1A* mutations [155,156], possibly reflecting different age-related penetrance; this probably also correlates with a higher risk of gastric cancer development. In relation to small bowel cancer development, no genotype-phenotype correlation in JPS has been demonstrated.

*Cowden Syndrome*—CS, an inherited autosomal dominant syndrome, has an estimated incidence of 1:250,000 [154] and a high age-related penetrance (about 80%) [157]. In 90% of cases, symptoms appear within the second decade, and are mainly represented by muco-cutaneous lesions including papillomas, trichilemmomas, acral keratosis, and multiple gastrointestinal polyps of various type [158]. CS predisposes to a high risk for breast, thyroid, endometrial, colorectal, kidney neoplasms and melanoma [159]. Small bowel cancer risk is undefined and only few case reports are present in the international literature [160,161]. Germline mutations in *PTEN*, located on 10q22–q23, are demonstrated in up to 80% of patients [154]. The *PTEN* gene encodes for phosphatidylinositol-3,4,5-trisphosphate 3-phosphatase, a protein involved in numerous cell functions and related to the mTOR pathway by downregulating PI3K signaling. All types of mutations can affect *PTEN* gene, mainly in exons 5, 7, and 8; nonsense mutations correlate with colorectal cancer [159].

## 7. Duodenal Neuroendocrine Lesions in Multiple Endocrine Neoplasia Type 1 (MEN1)

Duodenal neuroendocrine neoplasms represent a relatively uncommon, heterogeneous group of lesions, arising in the duodenum and in the major and minor ampullary regions [162,163]. The most recent WHO classification [164] recognizes three different entities, namely well-differentiated neuroendocrine tumors (NETs—grade 1, 2, or 3 according to proliferation), gangliocytic paragangliomas, and poorly differentiated neuroendocrine carcinomas (NECs).

Well differentiated duodenal NETs have been further distinguished in clinico-pathologic subtypes [162], including: gastrinomas (functioning gastrin producing NETs) which may be sporadic or MEN1/Zollinger-Ellison syndrome (ZES) associated; ampullary-type somatostatin-producing NETs which may be associated with MEN1 or Neurofibromatosis type 1; non-functioning NETs.

While the molecular alterations in precursor lesions of non-functioning NETs, NECs and gangliocytic paragangliomas are not well-defined, a little more information is available for MEN1 syndrome associated gastrinoma and somatostatinoma precursor lesions.

MEN1 patients harbor germline mutations of *MEN1* gene (11q13) [165], with either a loss of heterozygosity (LOH) in the gene and/or the centromere 11 [166] or as small intragenic somatic mutations.

With regards to MEN1-associated duodenal functioning gastrinomas, these are often multiple and associated with diffuse gastrin cell (G cell) hyperplasia (simple, linear, micronodular or macronodular neuroendocrine cell hyperplasia) and multicentric gastrin-producing microtumours (between 300 microns and 2 mm), which represent probable precursor lesions [167] (Figure 3D). These hyperplastic lesions have been identified in most subjects affected by MEN1, while are lacking in those with sporadic duodenal gastrinomas and ZES.

While approximately 50% of MEN1-associated duodenal NETs and microtumors show LOH on chromosome 11q13, the hyperplastic G cells consistently lack this finding [166]. This is important as it suggests that hyperplastic cells, though they harbor *MEN1* germline mutations, are not yet committed to neoplastic transformation and indeed, LOH on chromosome 11q13 is a crucial event in the etiopathogenesis of duodenal gastrinomas. Perhaps, the enhanced proliferation of G cells, which leads to hyperplasia, could be secondary to an increased responsiveness of germline mutated G cells to, as yet, unknown growth factors [168].

A second important aspect is that, as synchronous MEN1 tumors (regardless of size) show distinct deletion patterns, every gastrin-secreting neoplasm in a MEN-1 patient probably derives from independent clones and from an independent second hit.

Little is known about precursor lesions of ampullary-type somatostatin-producing NETs; however, scattered somatostatin cells forming linear or micronodular growths have been described in the normal ampullary epithelium adjacent to neoplasms [162,166]. Allelic loss (LOH at 11q13) has also been detected in duodenal somatostatin, producing MEN1-associated tumors as small as 400 microns; the hyperplastic somatostatin cells, however, similarly to the hyperplastic G-cells, do not show chromosome 11q13 LOH.

## 8. Conclusions

In this review, the relevant molecular heterogeneity of the epithelial preinvasive lesions of the small bowel is highlighted (Table 1). The management of such entities is based on an integrated diagnostic approach, requiring an accurate morphological, immunophenotypic and molecular/cytogenetic characterization and the use of advanced endoscopic (e.g., video capsule endoscopy, device assisted enteroscopy) or imaging procedures (e.g., computed tomography enteroclysis/enterography, magnetic resonance enteroclysis/enterography). Small bowel precursor lesion surveillance in high-risk groups is crucial as it can result in early disease detection. The growing awareness about the molecular basis underlying the pathogenesis of small bowel neoplastic epithelial lesions and the transposition of these data in the clinical practice will hopefully reduce cancer burden. In particular, the identification of some molecular alterations and dysregulated pathways found in such lesions as potential therapeutic targets opens up new horizons for the management of these disorders.

## Figures and Tables

**Table 1:** Main genetic alterations found in small bowel precursor epithelial lesions.

Subtype		Gene (Encoded Protein)	Prevalence of Mutations	Functional Effect
**Sporadic, non-ampullary, intestinal-type adenomas**		*APC* (Adenomatous polyposis coli protein)	50–55% [15,16]	Regulation of Wnt signaling pathway, cell migration and adhesion, apoptosis
		*KRAS* (KRas)	5–18% [15,16]	GTPase intracellular signal transducer, regulating proliferation and differentiation
		*BRAF* (BRaf)	0–4% [16]	Activation of the MAP kinase transduction pathway
		*ERBB2/HER2* (erbB2)	<5% [16]	Protein tyrosine kinase involved in stabilization of peripheral microtubules and transcriptional regulation
		*TP53* (p53)	<5% [15,16]	Regulation of cell cycle arrest, apoptosis, senescence and DNA repair
**Pyloric gland adenomas**		*GNAS* (G-alpha subunits of G proteins)	40% [27]	GPCR-mediated signaling constitutively active; PKA activation
**Foveolar adenomas**		*GNAS* (G-alpha subunits of G proteins)	100% [27]	GPCR-mediated signaling constitutively active; PKA activation
**Sporadic, ampullary, intestinal-type adenomas**		*APC* (Adenomatous polyposis coli protein)	17–44% [15,52]	Regulation of Wnt signaling pathway, cell migration and adhesion, apoptosis
		*KRAS* (KRas)	30–44% [15,55,56]	GTPase, intracellular signal transducer, regulating proliferation and differentiation
**Syndromic intestinal-type adenomas**	FAP	*APC* (Adenomatous polyposis coli protein)	17–66% [15,52,96]	Regulation of Wnt signaling pathway, cell migration and adhesion, apoptosis
		*KRAS* (KRas)	10% [96]	GTPase, intracellular signal transducer, regulating proliferation and differentiation
	MAP	*MUTYH* (Adenine DNA glycosilase)	100%	Oxidative DNA damage repair (base excision repair)
		*APC* (Adenomatous polyposis coli protein)	77% [96]	Regulation of Wnt signaling pathway, cell migration and adhesion, apoptosis
		*KRAS* (KRas)	33% [96]	GTPase, intracellular signal transducer, regulating proliferation and differentiation
**Crohn’s disease-associated dysplasia**		*KRAS* (KRas)	15–40% [113,115]	GTPase, intracellular signal transducer, regulating proliferation and differentiation
		*PIK3CA* (Phosphatidylinositol 4,5-biphosphate 3-kinase catalytic subunit alpha isoform)	0–60% [113,115]	Activation of cell signaling regulating cellular growth, proliferation and morphology
**Peutz-Jeghers polyps**		*STK11* (STK11)	>90% [143]	Tumor suppressor serine/threonine-protein kinase, controlling AMPK family members
**Juvenile polyps**		*SMAD4* (Smad4/Dpc4)	20% [151]	Tumor suppressor, mediator of signal transduction by TGF β
		*BMPR1A* (Bone morphogenetic protein receptor type-1A)	30% [151]	Transmembrane serine/threonine kinases, activation of SMAD transcriptional regulators
**Cowden syndrome polyps**		*PTEN* (Phosphatidylinositol 3,4,5-trisphosphate 3-phosphatase)	80% [154]	Tumor suppressor related to the mTOR pathway through downregulation of the PI3K signaling pathway

## Data Availability

No new data were created or analyzed in this study. Data sharing is not applicable to this article.
